# Supplementary material for: Exposure to Zika and chikungunya viruses impacts aspects of the vectorial capacity of Aedes aegypti and Culex quinquefasciatus
Source: PLoS One. 2024 May 15;19(5):e0281851. doi: 10.1371/journal.pone.0281851 (PMC11095752; doi:10.1371/journal.pone.0281851)

**Supporting information**

**S1 Table. Viral load (number of RNA copies of ZIKV – CN, per mL, detected by RT-qPCR), in *Aedes aegypti* colonies from the laboratory - RecL and field - AeCamp, infected with Zika virus (ZIKV), which completed blood meal on the 7 day post-exposure (dpe), in blood free of viral particles, as a function of post-exposure life span to the virus.**

|  | N | Min | Max | Mean | Median | Standard deviation | EP | **p-value** |
| --- | --- | --- | --- | --- | --- | --- | --- | --- |
| **RecL** |  |  |  |  |  |  |  |  |
| < 22 Rep - | 15 | 640 | 8.23E+07 | 6.13E+06 | 5.96E+03 | 2.11E+07 | 5.45E+06 | **0.0033** |
| < 22 Rep + | 22 | 900 | 5.07E+07 | 8.26E+06 | 4.05E+06 | 1.31E+07 | 2.79E+06 |  |
| ≥ 22 Rep - | 5 | 434 | 2.59E+06 | 5.49E+05 | 2.48E+03 | 1.14E+06 | 5.11E+05 | 0.5194 |
| ≥ 22 Rep + | 8 | 812 | 1.30E+07 | 2.69E+06 | 1.66E+05 | 4.82E+06 | 1.70E+06 |  |
| **Aecamp** |  |  |  |  |  |  |  |  |
| < 22 Rep - | 24 | 289 | 1.95E+07 | 2.52E+06 | 1.15E+06 | 4.32E+06 | 8.82E+05 | 0.0748 |
| < 22 Rep + | 13 | 2960 | 4.42E+07 | 1.09E+07 | 5.04E+06 | 1.43E+07 | 3.97E+06 |  |

Source: Fiocruz/PE. IAM's Statistics and Geoprocessing Center.

**S2 Table. Risk of death for females of *Ae. aegypti*, RecL and AeCamp colonies, after exposure to Zika virus (ZIKV).**

| **Variables** | **RecL** | | | | **AeCamp** | | | |
| --- | --- | --- | --- | --- | --- | --- | --- | --- |
|  | **HR** | **95% CI** | | **p value** | **HR** | **95% CI** | | **p value** |
|  |  | **info** | **top** |  |  | **info** | **top** |  |
| Not exposed Group | 1.000 |  |  |  | 1.000 |  |  |  |
| Exposed Group | 1.845 | 1.130 | 3.013 | 0.014 | 1.289 | 0.8165 | 2.034 | 0.276 |
| Exposed infected Group | 2014 | 1.272 | 3.189 | 0.003 | 1.212 | 0.7696 | 1.910 | 0.406 |
| **Variables** | **Proportionality** | | | | | | | |
|  |  | **Schoenfeld analysis** | | **p value** |  | **Schoenfeld analysis** | | **p value** |
|  |  | **Chi-square** | |  |  | **Chi-square** | |  |
| Exposed |  | 3.30 | | 0.070 |  | 0.112 | | 0.738 |
| Exposed Infected |  | 3.36 | | 0.067 |  | 1.142 | | 0.285 |
| **GLOBAL** |  | 4.87 | | 0.088 |  | 1.582 | | 0.453 |

Source: Fiocruz/PE. IAM's Statistics and Geoprocessing Center.

**S3 Table. Risk of death of females from the laboratory *Culex quinquefasciatus* colony – CqSLab, after exposure to the Zika virus (ZIKV).**

| **Variables** | **Hz** | **Survival** | | **p-value** |
| --- | --- | --- | --- | --- |
|  |  | **IC 95%** | |  |
|  |  | **Inf** | **Sup** |  |
| Control group | 1.000 |  |  |  |
| Exposed Group | 1.300 | 0.903 | 1.871 | **0.158** |
| Exposed Group I | 0.805 | 0.473 | 1.368 | **0.422** |
| **Variables** |  | **Proportionality** | | **p-value** |
|  |  | **Schoenfeld analysis** | |  |
|  |  | **Chi-square** | |  |
|  |  |  |  |  |
| Group |  | 0.439 | | **0.800** |
|  |  |  |  |  |
| **GLOBAL** |  | 0.439 | |  |

Source: Fiocruz/PE. IAM's Statistics and Geoprocessing Center.

**S4 Table. Risk of death for females from the laboratory *Aedes aegypti colony* – RecL after exposure to chikungunya virus (CHIKV) during the first 20 days of observation.**

| **Variables** | **HR** | **survival** | | **p-value** |
| --- | --- | --- | --- | --- |
|  |  | **95% CI** | |  |
|  |  | **info** | **top** |  |
| Not exposed Group | 1.000 |  |  |  |
| Exposed Infected | 3.963 | 1.73 | 9.08 | 0.001 |
| **Variables** |  | **Proportionality** | | **p-value** |
|  |  | **Schoenfeld analysis** | |  |
|  |  | **Chi-square** | |  |
| Exposed Infected |  | 0.0647 | | 0.799 |

Source: Fiocruz/PE. IAM's Statistics and Geoprocessing Center.

**S5 Table. Risk of death for females from the field colony of *Aedes aegypti* – AeCamp after exposure to chikungunya virus (CHIKV).**

| **Variables** | **HR** | **survival** | | **p-value** |
| --- | --- | --- | --- | --- |
|  |  | **95% CI** | |  |
|  |  | **info** | **top** |  |
|  |  |  |  |  |
| Not exposed Group | 1.000 |  |  |  |
| Exposed infected Group | 1.078 | 0.7092 | 1.638 | 0.725 |
| **Variables** |  | **Proportionality** | | **p-value** |
|  |  | **Schoenfeld analysis** | |  |
|  |  | **Chi-square** | |  |
| Exposed Infected |  | 0.0048 | | 0.945 |

Source: Fiocruz/PE. IAM's Statistics and Geoprocessing Center.

**S6 Table. Correlation between RNA copy number (CN), detected by RT-qPCR, and longevity, fecundity and fertility of females infected with Zika virus (ZIKV) or chikungunya virus (CHIKV).**

| **Virus/colonies** | | **Variables** | **rho** | **p value** | **p value** |
| --- | --- | --- | --- | --- | --- |
| **ZIKV** | **AeCamp** | **CN** | *** | **< 0.001** | *** |
|  |  | Longevity * | -0.505 | **0.011** | 0.166 |
|  |  | Fertility | -0.121 | 0.269 | 0.364 |
|  |  | Fecundity | -0.088 | 0.696 | 0.510 |
|  | **RecL** | **CN** | *** | **< 0.001** | *** |
|  |  | Longevity * | 0.232 | **0.005** | 0.265 |
|  |  | Fertility | 0.057 | **< 0.001** | 0.623 |
|  |  | Fecundity | -0.008 | 0.068 | 0.947 |
|  | **CqSLab** | **CN** | *** | **< 0.001** | *** |
|  |  | Longevity * | 0.207 | **< 0.001** | 0.458 |
|  |  | Fertility | 0.364 | **< 0.001** | 0.182 |
|  |  | Fecundity | 0.168 | **< 0.001** | 0.549 |
| **CHIKV** | **AeCamp** | **CN** | *** | **< 0.001** | *** |
|  |  | Longevity * | -0.534 | **0.004** | **0.033** |
|  |  | Fertility | 0.013 | **< 0.001** | 0.928 |
|  |  | Fecundity | 0.101 | 0.002 | 0.437 |
|  | **RecL** | **CN** | *** | **< 0.001** | *** |
|  |  | Longevity * | 0.310 | **< 0.001** | 0.184 |
|  |  | Fertility | -0.388 | **0.026** | **0.003** |
|  |  | Fecundity | 0.332 | **0.027** | **0.011** |

Source: Fiocruz/PE. IAM's Statistics and Geoprocessing Center. *Only mosquitoes that were not censored. P value^1^ - Evaluates data normality / P value^2^ - Evaluates data correlation

**S7 Table. Mean and median numbers of eggs laid and percentage of eggs that hatched in the first gonotrophic cycle (GC) of *Ae. aegypti* – RecL and AeCamp colonies, and *Culex quinquefasciatus* – CqSLab colony – after exposure to the Zika virus (ZIKV).**

| **Colony** | **Groups** | **Fecundity (number of eggs)** | | | | | **Fertility (percentage of eggs that hatched)** | | | | |
| --- | --- | --- | --- | --- | --- | --- | --- | --- | --- | --- | --- |
|  |  | **N** | Confidence Interval | Mean | Median | p value | **N** | Confidence Interval | Mean | Median | p value |
| RecL | Control | 149 | 82.95 – 92.71 | 87.83 | 88.00 | **0.1540**^2^ | 149 | 63.90 - 70.82 | 67.36 | 67.96 | 0.7640^2^ |
|  | Exposed | 46 | 70.02 – 88.24 | 79.13 | 74.00 |  | 46 | 59.25 – 73.37 | 66.31 | 74.38 |  |
|  | Exposed infected | 83 | 75.42 – 88.72 | 82.07 | 89.00 |  | 82 | 63.96 – 74.28 | 69.12 | 73.64 |  |
|  | **Significance** |  | | | | |  | | | | |
| AeCamp | Control | 150 | 67.93 – 77.48 | 72.70 | 75.00 | **0.8420**^2^ | 150 | 62.22 – 68.71 | 65.46 | 65.25 | **< 0.0001**^2^ |
|  | Exposed | 50 | 61.97 – 78.79 | 70.40 | 70.00 |  | 50 | 44.78 – 56.58 | 50.68 | 51.30 |  |
|  | Exposed infected | 76 | 64.99 – 76.85 | 70.90 | 71.50 |  | 76 | 45.45 – 53.30 | 49.38 | 50.63 |  |
|  | **Significance** | control x exposed p **< 0,0001**; control x exposed infected p **< 0,0001** | | | | | | | | | |
| CqSLab | Control | 60 | 95.12 – 109.38 | 102.25 | 105.00 | **< 0.0001**^2^ | 59 | 74.91 – 81.99 | 78.45 | 81.13 | 0.5888^1^ |
|  | Exposed | 61 | 68.09 – 85.71 | 76.90 | 85.00 |  | 56 | 73.03 – 80.50 | 76.77 | 77.71 |  |
|  | Exposed infected | 18 | 81.19 – 104.14 | 92.67 | 94.50 |  | 18 | 77.28 – 84.63 | 80.96 | 82.54 |  |
|  | **significance** | control x exposed **p<0,0001** | | | | | - | | | | |

Source: Fiocruz/PE. IAM's Statistics and Geoprocessing Center.

Kruskal-Wallis ^1^ / Anova ^2^

**S8 Table. Mean and median number of eggs laid and mean and median percentage of eggs that hatched, in the first gonotrophic cycle of *Ae. aegypti* – RecL and AeCamp colonies, after exposure to chikungunya virus (CHIKV).**

| **Colony** | | | **Groups** |  | | | | |
| --- | --- | --- | --- | --- | --- | --- | --- | --- |
|  |  |  |  | **N** | **Confidence Interval** | **Mean** | **Median** | **p value** |
| **Fecundity** | **(number of eggs)** | RecL | Control | 99 | 60.87 – 71.48 | 66.17 | 68.00 | **0.0690 ^1^** |
|  |  |  | Exposed infected | 98 | 53.23 – 64.72 | 58.98 | 61.00 |  |
|  |  | AeCamp | Control | 100 | 47.00 – 55.50 | 50.56 | 48.00 | **< 0.0001 ^2^** |
|  |  |  | Exposed infected | 94 | 36.50 – 45.50 | 39.60 | 38.00 |  |
| **Fertility** | **(percentage of eggs that hatched)** | RecL | Control | 98 | 55.56 – 64.23 | 59.89 | 63.48 | **< 0.0001^2^** |
|  |  |  | Exposed infected | 94 | 37.62 – 45.51 | 41.57 | 40.67 |  |
|  |  | AeCamp | Control | 97 | 51.01 – 58.32 | 54.66 | 57.50 | **< 0.0001^2^** |
|  |  |  | Exposed infected | 87 | 34.50 – 41.51 | 38.01 | 37.50 |  |

Source: Fiocruz/PE. IAM’s Statistics and Geoprocessing Center.

T-Student Test ^1^ / Mann-Whitney ^2^

**S1 Fig. Mean number of RNA copies (CN) detected at periods ≤7 days; 8 to 14 and 15 to 21 days post-exposure - DPE to Zika virus ZIKV, in colonies of *Aedes aegypti* in the field (AeCamp), in laboratory (RecL).**


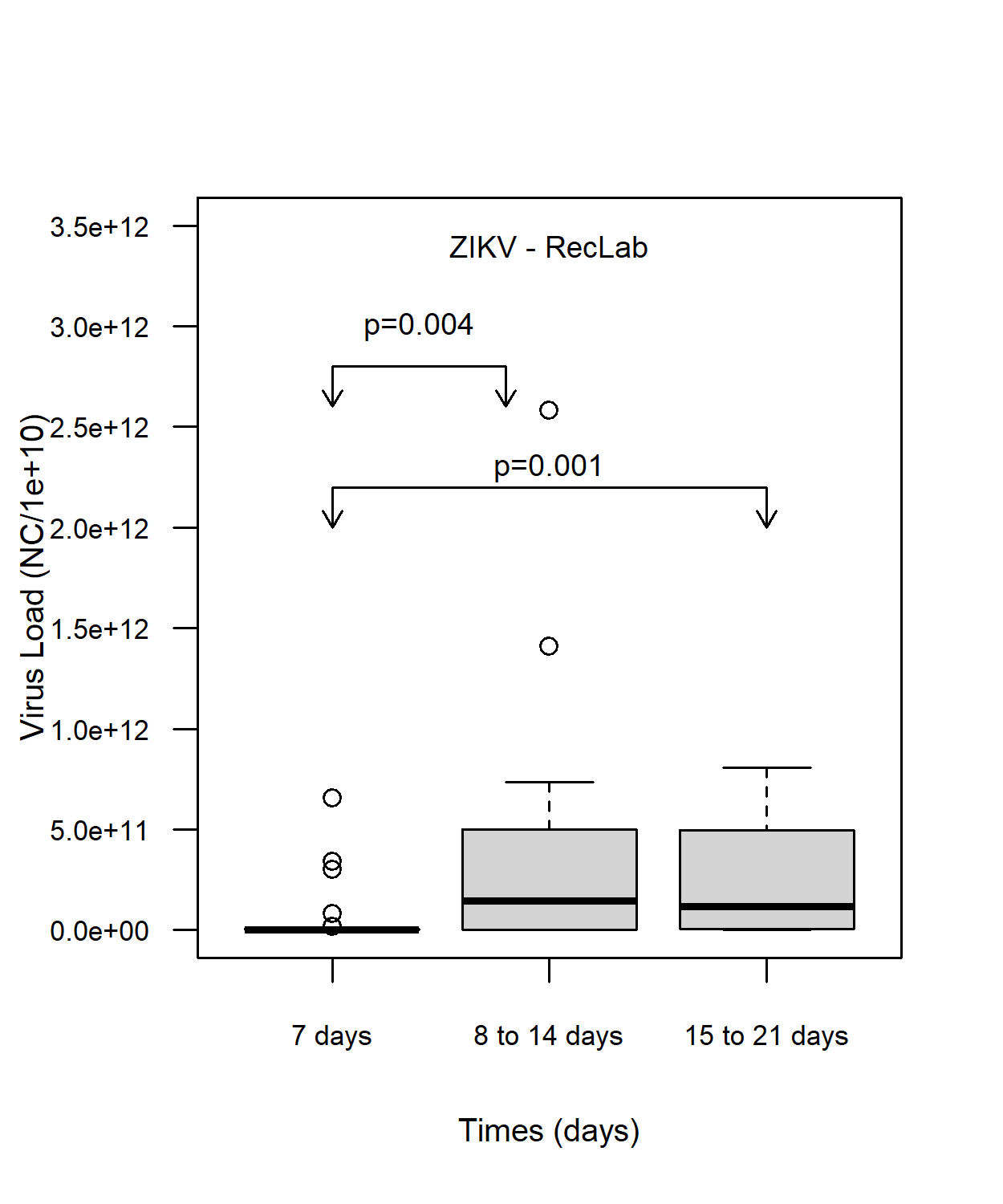

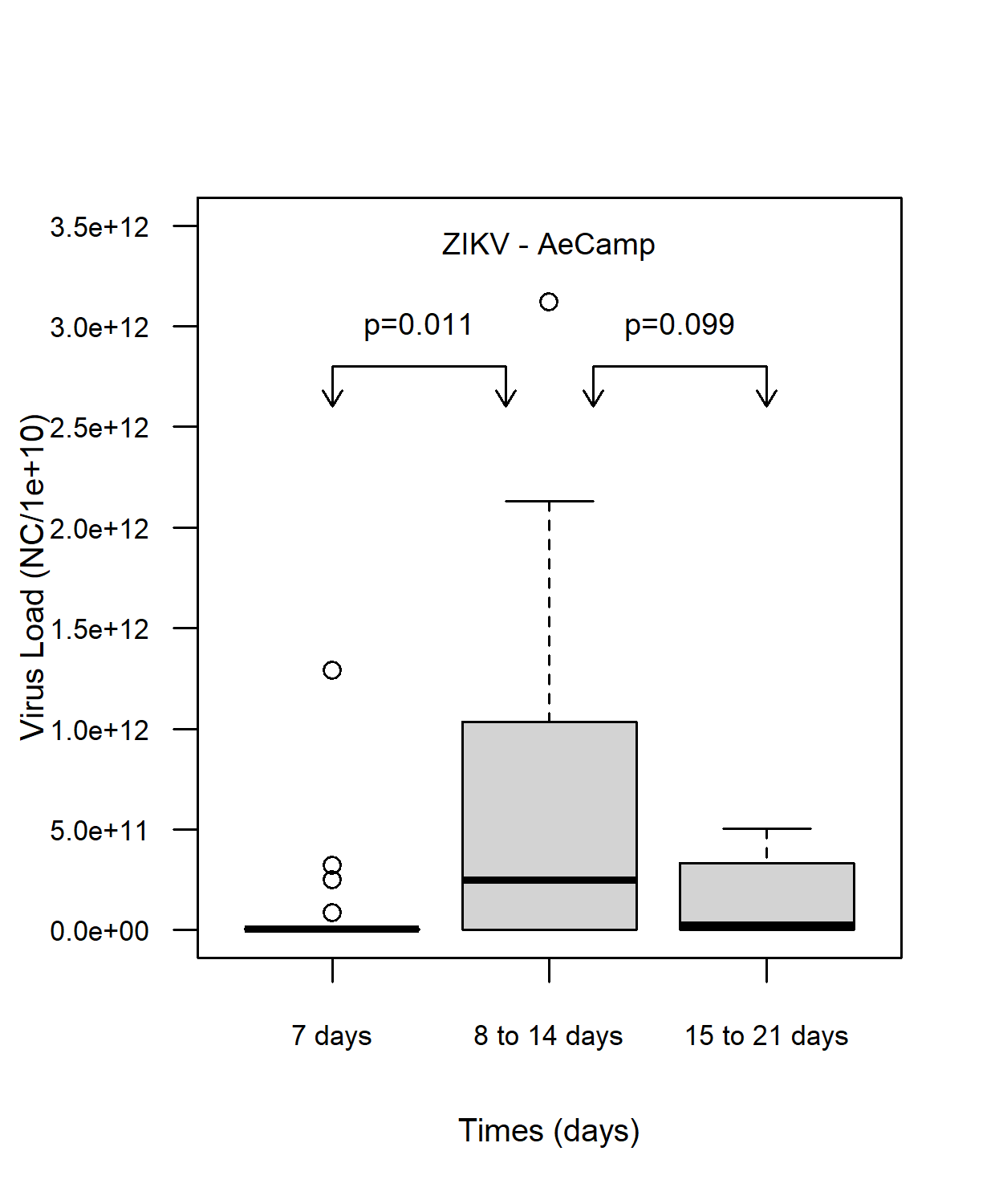

Supplement: S1 File — (DOCX) [file pone.0281851.s001.docx]
