# Supplementary material for: Exposure to Zika and chikungunya viruses impacts aspects of the vectorial capacity of Aedes aegypti and Culex quinquefasciatus
Source: PLoS One. 2024 May 15;19(5):e0281851. doi: 10.1371/journal.pone.0281851 (PMC11095752; doi:10.1371/journal.pone.0281851)
Supplement: Attachment — Submitted filename: Review.docx [file pone.0281851.s003.docx]

Peer Review for:

**Manuscript Number:** PONE-D-23-02967
**Manuscript Title:** Exposure to Zika and chikungunya viruses impacts aspects of the vectorial capacity of Aedes aegypti and Culex quinquefasciatus

In their manuscript Crespo et al explore the impact of ZIKV and CHIKV arbovirus exposure and infection on reproductive capacity, longevity, and blood feeding of *Aedes aegypti* and *Culex quinquefasciatus* mosquitoes. They accomplish this by exposing lab and field-caught colonies of *Ae. aegypti* to both ZIKV and CHIKV, and a lab colony of *Cx. quinquefasciatus* to ZIKV and then assessing fecundity and fertility. To evaluate survival and longevity, the authors collected *Ae. aegypti* mosquitoes at 7, 14, and 21 days post exposure, but only collected *Cx. quinquefasciatus* when death occurred throughout the experiment. FTA cards infused with Manuka honey blend were utilized for viral detection. Additionally, blood-seeking behavior was assessed at 7, 14, and 21 days post exposure. RT-qPCR was performed for viral detection of the samples. While it’s significant that both viruses were associated with a biological cost, in particular, diminished reproductive capability, the fact that blood feeding activity remained the same (or even increased) after exposure helps further explain viral dissemination and global expansion of these arboviruses.

Major Issues:

Line 170-171 - Why were *Cx. quinquefasciatus* mosquitoes treated differently (evaluated differently) from *Ae. aegypti*?

Line 244-247 - More information is needed concerning the contamination of samples and inability to carry out 2^nd^ and 3^rd^ experiments. Did you consider re-running the experiment for the sake of replication?

Figures 1-3 are not visible on the downloaded PDF.

Minor Issues:

Please see my edited version of your manuscript with highlights and comments.

Line 28 – no period is needed after “AeCamp”

Line 38-39 – consider defining “vectorial capacity” a bit here. You may also consider differentiating “vectorial capacity” from “vector competence”

Line 41-43 – The cited study involves *Anopheles* mosquitoes and doesn’t relate well as an “example” of what you previously stated.

Line 67 – “vector” should read “vectorial”

Line 68-70 – consider re-wording this sentence as it is hard to understand

Line 71 – use commas instead of semicolons.

Line 83-84 – This could use a reference.

Line 100 – What is “shelling”?

Line 113 – “tittered” should be “titered”

Line 181 – Consider including the timeframe that mosquitoes were allowed to seek a blood meal.

For Figure captions, please include a clear description of what group each panel represents. For example, you do this in Fig 5 caption (A: RecL Colony; B: AeCamp Colony), but not clearly for Fig 4.

Line 359 – Consider “blood feeding” instead of “blood meal activity”

Line 377 – remove the “a”.

Line 494 – Consider changing “of control of” to “to control”

Overall, this is a well designed and executed study to further explore the biological cost of arbovirus exposure and the impact of infection on vectorial capacity. Recommend acceptance for publication after minor revisions.
