# Supplementary material for: Exposure to Zika and chikungunya viruses impacts aspects of the vectorial capacity of Aedes aegypti and Culex quinquefasciatus
Source: PLoS One. 2024 May 15;19(5):e0281851. doi: 10.1371/journal.pone.0281851 (PMC11095752; doi:10.1371/journal.pone.0281851)
Supplement: Attachment — Submitted filename: Review of Revision_Reviewer 2.docx [file pone.0281851.s005.docx]

Review for Revision 1 of:

**Manuscript Number:** PONE-D-23-02967
**Manuscript Title:** Exposure to Zika and chikungunya viruses impacts aspects of the vectorial capacity of Aedes aegypti and Culex quinquefasciatus

Additional Minor Issues:

Line 50 – Add space before “Modifications”

Line 54 – Flaviviridae and Togaviridae should both be *italicized* ( *Flaviviridae and Togaviridae* )

Line 88 – Add a period.

In the “Study Area” section, consider adding avg. annual precipitation.

Line 92 – remove “and”

Line 102 – delete duplicate “and”

Line 103 – delete space and 1 “.” after the word “dipper”

Line 167 – use a comma, not a semicolon as in line 173.

Line 231 – no ‘ needed after “Wallis”

Table 2 is difficult to interpret; data doesn’t seem to fit correctly. Be consistent. Some numbers use “e” others “E” and 2 of the numbers just have a long string of integers.

Consider just using decimal points for the numbers in your tables, not commas.

Line 359 – 360 – delete the space between 63.48 and the “%” as well as with 40.67

Line 363 – add % after 57.50 and 37.50

Line 400 – delete extra comma after “6”

Line 460 – I believe the word you intend, throughout the paper is “gonotrophic” as opposed to gonadotropic or gonadotrophic. Consider using gonotrophic at line 134, 157, 162, 168, Fig 4 legend, 370, and 460.

Line 540 – It is worth highlighting…

Line 543 – “pointed out” of better, “discussed” or “elucidated”
