# Supplementary material for: Exposure to Zika and chikungunya viruses impacts aspects of the vectorial capacity of Aedes aegypti and Culex quinquefasciatus
Source: PLoS One. 2024 May 15;19(5):e0281851. doi: 10.1371/journal.pone.0281851 (PMC11095752; doi:10.1371/journal.pone.0281851)
Supplement: Attachment — Submitted filename: Response 2_PointbyPoint 12152023.docx [file pone.0281851.s006.docx]

Dear Kelli L. Barr,

Academic Editor PLOS ONE

Thanks for this review. Below are our responses to each question raised by reviewers

Revision 2 – article Crespo et al, 2023

**Comments to the Author**

1. If the authors have adequately addressed your comments raised in a previous round of review and you feel that this manuscript is now acceptable for publication, you may indicate that here to bypass the “Comments to the Author” section, enter your conflict of interest statement in the “Confidential to Editor” section, and submit your "Accept" recommendation.

Reviewer #1: All comments have been addressed

Reviewer #2: All comments have been addressed

Reviewer #3: (No Response)

2. Is the manuscript technically sound, and do the data support the conclusions?

The manuscript must describe a technically sound piece of scientific research with data that supports the conclusions. Experiments must have been conducted rigorously, with appropriate controls, replication, and sample sizes. The conclusions must be drawn appropriately based on the data presented.

Reviewer #1: Partly

Reviewer #2: Yes

Reviewer #3: Yes

3. Has the statistical analysis been performed appropriately and rigorously?

Reviewer #1: Yes

Reviewer #2: Yes

Reviewer #3: Yes

4. Have the authors made all data underlying the findings in their manuscript fully available?

The [PLOS Data policy](http://www.plosone.org/static/policies.action#sharing) requires authors to make all data underlying the findings described in their manuscript fully available without restriction, with rare exception (please refer to the Data Availability Statement in the manuscript PDF file). The data should be provided as part of the manuscript or its supporting information, or deposited to a public repository. For example, in addition to summary statistics, the data points behind means, medians and variance measures should be available. If there are restrictions on publicly sharing data—e.g. participant privacy or use of data from a third party—those must be specified.

Reviewer #1: Yes

Reviewer #2: Yes

Reviewer #3: Yes

5. Is the manuscript presented in an intelligible fashion and written in standard English?

PLOS ONE does not copyedit accepted manuscripts, so the language in submitted articles must be clear, correct, and unambiguous. Any typographical or grammatical errors should be corrected at revision, so please note any specific errors here.

Reviewer #1: Yes

Reviewer #2: Yes

Reviewer #3: No

6. Review Comments to the Author

Please use the space provided to explain your answers to the questions above. You may also include additional comments for the author, including concerns about dual publication, research ethics, or publication ethics. (Please upload your review as an attachment if it exceeds 20,000 characters)

**Reviewer #2: Review for Revision 1 of:**
Manuscript Number: PONE-D-23-02967
Manuscript Title: Exposure to Zika and chikungunya viruses impacts aspects of the vectorial capacity of Aedes aegypti and Culex quinquefasciatus

Additional Minor Issues:
Line 50 – Add space before “Modifications”

**Author's response to reviewer: done**

Line 54 – Flaviviridae and Togaviridae should both be italicized (Flaviviridae and Togaviridae )

**Author's response to reviewer: done**

Line 88 – Add a period.

**Author's response to reviewer: done**

In the “Study Area” section, consider adding avg. annual precipitation.

**Author's response to reviewer: done**

Line 92 – remove “and”

**Author's response to reviewer: done**

Line 102 – delete duplicate “and”

**Author's response to reviewer: done**

Line 103 – delete space and 1 “.” after the word “dipper”

**Author's response to reviewer: done**

Line 167 – use a comma, not a semicolon as in line 173.

**Author's response to reviewer: done**

Line 231 – no ‘ needed after “Wallis”

**Author's response to reviewer: done**

Table 2 is difficult to interpret; data doesn’t seem to fit correctly. Be consistent. Some numbers use “e” others “E” and 2 of the numbers just have a long string of integers.
Consider just using decimal points for the numbers in your tables, not commas.

**Author's response to reviewer: done**

Line 359 – 360 – delete the space between 63.48 and the “%” as well as with 40.67

**Author's response to reviewer: done**

Line 363 – add % after 57.50 and 37.50

**Author's response to reviewer: done**

Line 400 – delete extra comma after “6”

**Author's response to reviewer: done**

Line 460 – I believe the word you intend, throughout the paper is “gonotrophic” as opposed to gonadotropic or gonadotrophic. Consider using gonotrophic at line 134, 157, 162, 168, Fig 4 legend, 370, and 460.

**Author's response to reviewer: done**

Line 540 – It is worth highlighting…

**Author's response to reviewer: done**

Line 543 – “pointed out” of better, “discussed” or “elucidated”

**Author's response to reviewer: done**

**Reviewer #3:**

The manuscript has improved after the suggestions made by the reviewers, and I thank the authors for that. However, I still have some considerations about the data and the conclusions made.
The authors wrote in the Discussion section: “In summary, despite the significant reduction in some aspects of the biological performance of Ae. aegypti, for both viruses and for Cx. quinquefasciatus, infected with ZIKV, our data suggest that their vectorial capacity is probably not affected, as the longevity and feeding behavior of field mosquitoes were not impacted by virus.” They also mention that the data presented in the manuscript corroborated other published data. Were the authors expecting any different results? Why or why not? Would you expect to find any differences in mosquitoes from the field? If so, do you have any hypothesis to explain why it did not happen? Sorry if I am being repetitive, but I still miss some sentences describing the new relevant data of this manuscript.

**Author's response to reviewer: We changed the last paragraph of the discussion to clarify these issues.**
